# Seroepidemiological study on the spread of SARS-CoV-2 in Germany: Study protocol of the CORONA-MONITORING bundesweit’ study (RKI-SOEP study)

**DOI:** 10.25646/7853

**Published:** 2021-03-25

**Authors:** Jens Hoebel, Markus A. Busch, Markus M. Grabka, Sabine Zinn, Jennifer Allen, Antje Göfêwald, Jörg Wernitz, Jan Goebel, Hans Walter Steinhauer, Rainer Siegers, Carsten Schroder, Tim Kuttig, Hans Butschalowsky, Martin Schlaud, Angelika Schaffrath Rosario, Jana Brix, Anna Rysina, Axel Glemser, Hannelore Neuhauser, Silke Stahlberg, Antje Kneuer, Isabell Hey, Jörg Schaarschmidt, Julia Fiebig, Nina Buttmann-Schweiger, Hendrik Wilking, Janine Michel, Andreas Nitsche, Lothar H. Wieler, Lars Schaade, Thomas Ziese, Stefan Liebig, Thomas Lampert

**Affiliations:** 1 Robert Koch Institute, Berlin Department of Epidemiology and Health Monitoring; 2 German Institute for Economic Research, Berlin, Socio-Economic Panel; 3 Humboldt University Berlin Faculty of Humanities and Social Sciences; 4 Freie Universität Berlin School of Business and Economics; 5 Kantar GmbH, Munich; 6 Robert Koch Institute, Berlin Department of Infectious Disease Epidemiology; 7 Robert Koch Institute, Berlin Centre for Biological Threats and Special Pathogens; 8 Robert Koch Institute, Berlin Institute Leadership; 9 Robert Koch Institute, Berlin Methodology and Research Infrastructure

**Keywords:** SARS-COV-2, COVID-19, SEROEPIDEMIOLOGICAL STUDY, CROSS-SECTIONAL STUDY, STUDY PROTOCOL

## Abstract

The SARS-CoV-2 coronavirus has spread rapidly across Germany. Infections are likely to be under-recorded in the notification data from local health authorities on laboratory-confirmed cases since SARS-CoV-2 infections can proceed with few symptoms and then often remain undetected. Seroepidemiological studies allow the estimation of the proportion in the population that has been infected with SARS-CoV-2 (seroprevalence) as well as the extent of undetected infections. The ‘CORONA-MONITORING bundesweit’ study (RKI-SOEP study) collects biospecimens and interview data in a nationwide population sample drawn from the German Socio-Economic Panel (SOEP).

Participants are sent materials to self-collect a dry blood sample of capillary blood from their finger and a swab sample from their mouth and nose, as well as a questionnaire. The samples returned are tested for SARS-CoV-2 IgG antibodies and SARS-CoV-2 RNA to identify past or present infections.

The methods applied enable the identification of SARS-CoV-2 infections, including those that previously went undetected. In addition, by linking the data collected with available SOEP data, the study has the potential to investigate social and health-related differences in infection status. Thus, the study contributes to an improved understanding of the extent of the epidemic in Germany, as well as identification of target groups for infection protection.

## 1. Introduction

The novel coronavirus SARS-CoV-2 (Severe Acute Respiratory Syndrome Coronavirus 2), which was first identified in the Chinese city of Wuhan in December 2019, has rapidly spread across the globe. The first cases of COVID-19, the disease caused by the virus, were reported in Germany at the end of January 2020 [1, 2]. Shortly thereafter, the virus had spread across Germany, so that by the beginning of March 2020, cases of COVID-19 had been recorded in all 16 federal states [3].

To contain the further spread of the virus, regulations on social distancing and movement outside of the home, severe restrictions on businesses as well as closures of childcare centres and schools have been imposed across Germany since mid-March 2020. After a peak of newly notified daily COVID-19 cases in March, infection numbers decreased considerably over the following weeks, enabling a gradual relaxation of containment measures from the end of April. The number of SARS-CoV-2 infections in Germany in the summer months was comparatively low [4]. From the end of September onwards, however, the number of cases significantly increased, with the total number of cases notified in Germany more than doubling between mid-August and the end of October [5, 6]. A total of 518,753 SARS-CoV-2 infections and cases of COVID-19 had been confirmed by laboratory diagnosis in Germany by 31 October 2020, corresponding to a cumulative incidence of 624 cases per 100,000 people [6]. New measures to contain the pandemic were subsequently introduced at the beginning of November 2020. People were asked to reduce their contact with those from other households to an absolute minimum. Meetings in public were severely restricted, and large parts of the hospitality industry, as well as leisure, sports and cultural facilities, were closed. Tourist accommodation and events for entertainment purposes were banned, although schools, childcare centres and shops initially remained open.

The figures and findings on the development of SARS-CoV-2 in Germany mentioned above are based on the statutory reporting of laboratory-confirmed cases of infection to health authorities. These data are collected nationwide according to the Protection against Infection Act (Infektionsschutzgesetz, IfSG) and compiled by the Robert Koch Institute (RKI). Since SARS-CoV-2 infections often remain undetected, for example, if a case remains unnoticed because of a lack of symptoms, it must be assumed that the incidence of infection is underrepresented in IfSG notification data. Population-based studies with serological detection of SARS-CoV-2 antibodies, on the other hand, enable the identification of past infections, including those that previously went undetected. For this reason, the World Health Organization recommends such seroepidemiological studies to improve the understanding of the spread of the virus in the population [7].

In spring 2020, the RKI thus began planning various serological studies to determine the proportion of the population with antibodies (seroprevalence) against the novel coronavirus. Serological testing of blood donations for SARS-CoV-2 antibodies (SeBluCo study), which the RKI is conducting in cooperation with blood donation services and virology institutes in 28 regions, began in April [8]. In May, data collection began for the ‘CORONA-MONITORING lokal’ study, in which the RKI has been testing population samples for SARS-CoV-2 antibodies as well as for current viral infection in four areas especially affected by the COVID-19 pandemic [9]. In addition, a study is being conducted in conjunction with the German Youth Institute to coincide with childcare centres being re-opened, which should provide insight into the role of preschool children in transmitting the disease [10]. The ‘CORONA-MONITORING bundesweit’ study (RKI-SOEP study) presented here focuses on the general population of Germany and has been developed by the RKI together with the German Institute for Economic Research (DIW). In this study, the research-based infrastructure of the German Socio-Economic Panel (SOEP) at the DIW is used to examine past and current infections with SARS-CoV-2 in people from all over Germany. It involves an analysis of IgG (immunoglobulin G) antibodies from self-collected capillary blood samples and viral RNA (ribonucleic acid) from oral-nasal swabs.

The SOEP is a multidisciplinary, long-term study that involves interviews of around 30,000 people from about 18,000 households throughout Germany every year [11]. An important aspect of this study is that the same people are interviewed every year. As a result, existing SOEP infrastructure can be used alongside a range of information on SOEP participants from previous survey waves, for example, information on living conditions, social situation and state of health, which can provide crucial information on the spread of SARS-CoV-2 in different population subgroups, and potentially reveal mechanisms involved in the spread of the virus [12].

The objectives of the ‘CORONA-MONITORING bundesweit’ study (RKI-SOEP study) are to analyse the following within a Germany-wide sample of the general adult population:

1. the seroprevalence, i.e., the proportion of the population in Germany with detectable IgG antibodies against SARS-CoV-2, differentiated by age group and sex,
2. the extent of undetected SARS-CoV-2 infections,
3. risk and protective factors for a SARS-CoV-2 infection, taking demographic, socioeconomic and health-related factors into account.

In addition, the RKI-SOEP study aims to lay the foundations for an analysis of the long-term effects of infection with SARS-CoV-2 over the course of people’s lives. Moreover, it can serve as a starting point for a Germany-wide analysis of developments over time in the spread of antibodies against SARS-CoV-2 at population level. The results of the study should also help to support the planning of non-pharmaceutical interventions to contain the COVID-19 pandemic and to develop targeted vaccination strategies.

## 2. Methodology

### 2.1 Study design and sampling

#### Study design

The ‘CORONA-MONITORING bundesweit’ study (RKI-SOEP study) is a population-based seroepidemiological observational study based on the DIW’s nationwide SOEP population samples. The SOEP is a longitudinal survey in Germany of private households and all persons living in them that has been conducted annually since 1984 [11]. The survey covers a wide range of topics, from demographics, income, the labour market, education and health through to people’s basic orientations, concerns and levels of satisfaction.

#### Sampling

The SOEP comprises a total of 25 different sub-samples made up of random samples of all private households in Germany, as well as samples from specific population groups such as migrants, refugees, families, high-income earners, households in socially disadvantaged neighbourhoods, homosexual and bisexual people, etc. [11]. The exact composition of samples, willingness to participate, dropout mechanisms and consequent changes to SOEP samples since the initial SOEP survey in 1984 are described in detail elsewhere [13]. Among SOEP participants, the willingness to participate in the SOEP again is generally high, at about 85%. Nevertheless, like all comparable survey studies of the general population, the SOEP does record selective panel attrition due to people’s refusal to participate again. SOEP compensates for panel attrition with regular refreshment samples and by making methodologically high-quality weighting factors available for population-related analyses (see also [13]).

The ‘CORONA-MONITORING bundesweit’ study (RKI-SOEP study) includes the SOEP core samples [14], the innovation sample [15] and the new migration samples (M1 and M2 [16, 17]). This renders a gross sample of a total of 31,675 persons aged 18 to 101 years from 19,574 households. This gross sample covers all 401 districts in Germany. There are districts with between one and 540 SOEP households. On average, the SOEP samples contacted for participating in the study contain 49 households per district with a standard deviation of 54 households. One SOEP household thereby includes between one and 12 persons, with an average of 1.6 persons aged 18 and over with a standard deviation of 0.7.

#### Inclusion and exclusion criteria

People are included who

▶ live in a SOEP household from one of the core samples, the innovation sample or the migration samples (M1 and M2)
▶ are at least 18 years old
▶ provide written consent to participate in the study
▶ are able to take a capillary blood sample and an oralnasal swab themselves (self-sampling).

People who lack the necessary German language skills to understand the German-language study material cannot successfully participate in the study.

### 2.2 Study implementation

#### Recruitment of participants and field control

All SOEP households from the gross sample receive a letter inviting them to participate in the study. The fieldwork is carried out by the field study institute Kantar GmbH based in Munich. Initially, all target households are sent a letter informing them that they will receive an invitation to participate in the study in the next few days. This letter includes a leaflet with information about the study and a letter from the German Federal Minister of Health encouraging participation. A few days later, each target person receives a letter inviting them to participate in the study. Target persons are defined as SOEP participants who were at least 18 years old as of 1 September 2020, who participated in the SOEP surveys in 2019 and/or 2020 and who have not explicitly stated that they do not wish to participate in future studies. These invitations contain both a personal invitation and study materials in German (data privacy statement, consent form, participation plan, short questionnaire, two specimen collection sets for self-sampling including instructions and packaging for posting the samples safely as well as two return envelopes: one for the consent form and short questionnaire, and one for the collected samples). As an incentive to participate in the study, potential participants are told that they will receive the laboratory results from the samples they collect, meaning that via the study they will find out their antibody and infection status. Participants who do not answer this letter receive a written reminder to participate in the study around two to three weeks after receiving the invitation.

The field phase started when the initial information letters were sent to target households on 30 September 2020. The first invitations including study materials were sent to each target person in the target households from 2 October 2020 onwards, the data collection phase therefore started at the beginning of October 2020 and is expected to continue until February 2021.

Due to the logistics of sampling, people in the gross sample were divided into four groups, which were invited to participate in the study one after the other. When creating the first three groups, stratification by federal states took into account that the measures taken to contain the spread of SARS-CoV-2 infections differed in part between federal states. In order to depict infection rates sufficiently accurately within the individual groups, households in the sample were also stratified in each federal state according to the reported cumulative incidence of infection at the district level (as of 14 September 2020). For this purpose, households in the RKI-SOEP gross sample in each federal state were assigned to one of three incidence categories based on the cumulative incidence per 100,000 inhabitants. Assignment to the low, medium or high incidence categories was based on terciles. From each of these three groups, households in the RKI-SOEP gross sample, minus the more recent SOEP migrant samples (M1 and M2), were randomly assigned to the first three groups in a ratio of 50%/25%/25%. The first group thus includes 14,535 adults and the two following groups 7,181 and 7,078 adults. The fourth group consists of people from the more recent SOEP migrant samples (M1 and M2) and comprises 2,881 adults. This fourth group was not stratified and was only used at the end of the field phase due to the greater administrative effort involved in using the M1 and M2 samples.

#### Data collection

The study consists of two parts: an examination and an interview. In the examination, participants take their own biological samples for a laboratory analysis of SARS-CoV-2 virus material. This determines their SARS-CoV-2 antibody status by means of ELISA (enzyme-linked immunosorbent assay), as well as current infection status by means of PCR (polymerase chain reaction; see also Chapter 2.3). In the interview, a short questionnaire is used to collect supplementary data on the participants.

**Figure 1:**
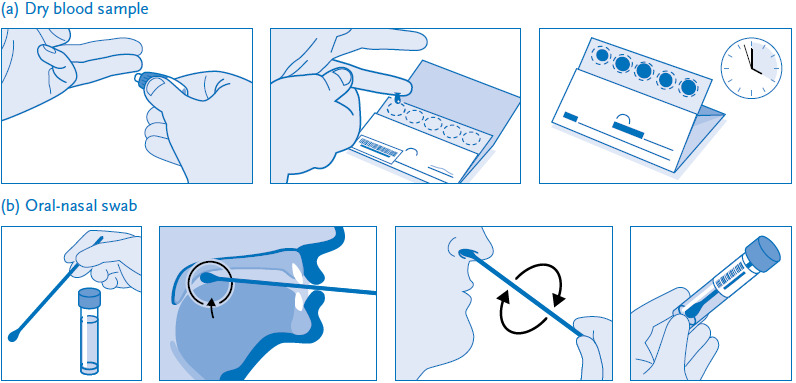
Self-collection of capillary blood from the fingertip (a) and a swab from the mouth and nose (b) Source: Own diagram

Participants are asked to provide a dry capillary blood sample from their fingertip for laboratory analysis of their antibody status. This can detect a previous infection with SARS-CoV-2. To test for a current infection with SARS-CoV-2, the participants are asked to provide a swab sample from their mouth and nose. Specimens are collected by the participants themselves (Figure 1) using CE-certified Euroimmun sample collection and submission kits (blood sample) and Copan eSwab collection systems (swab) sent by post. The blood sampling set contains detailed illustrated instructions with written explanations under each picture, a blood collection card, a compress, two plasters, two alcohol swabs, two sterile lancets and a sealable plastic bag containing a desiccant. There are five circles on the blood collection card to mark where blood should be collected. The collection and transport items supplied for the oral-nasal swab include a sample tube, a swab, a transport tube and a protective bag with an absorbent insert. The illustrated instructions in the specimen collection kit also include a web link and a QR code for access to supporting video instructions. In addition, there is a website specific to the study which has a list of frequently asked questions and answers (FAQ list). Study subjects are advised not to collect samples if there are any impediments or acute health conditions that would interfere with taking a sample or make it painful to do so.

Participants then send the collected biospecimens to the RKI in the appropriate enclosed envelope. Participants are requested to send specimens on the day of collection if at all possible. In order to assign test results to the personal data, which is stored at the Kantar field institute, and to link these results to the interview data, participants attach machine-readable barcodes to the blood collection cards, swab tubes, questionnaires and consent forms. These barcodes are included in the package in the form of adhesive labels. The questionnaires and consent forms, which must also have barcodes attached, are returned to Kantar together.

#### Communication of results

If the laboratory results do not require extra contact with study participants and are not subject to legal notification obligations, they are sent to participants in the form of individual result reports. For participants with questions about the results, there is the study-specific website with FAQ list, a form to contact the medical study staff, and a telephone hotline.

**Info box: Sensitivity and Specificity**

**Sensitivity** indicates the probability with which a test correctly identifies people with antibodies or a current infection.

**Specificity** indicates the probability with which a test correctly identifies people who do not have antibodies or are currently not infected.

In case of a positive PCR test, the relevant health authorities are notified within 24 hours and given the participant’s information. Immediately after notifying the local health authorities, the medical study staff send a written message to the person concerned with instructions on what to do. Whenever possible, the person who has tested positive is phoned by the medical study staff. Participants with a borderline SARS-CoV-2 PCR result are contacted in writing by the medical study staff within 72 hours of the result and are advised to repeat the PCR test at their local health authority. In addition, the medical study staff contact the responsible health authority by telephone and in writing.

#### Quality assurance

Quality assurance measures are integral to the entire study process and are implemented by all project partners (Kantar, RKI and the SOEP at the DIW). Field monitoring during the study and a weekly exchange with all project partners make it possible to react quickly to unexpected developments and take appropriate countermeasures (e.g. field time extensions to increase the response rate). Results from project meetings are recorded in writing and made available to the entire study staff. In addition, extensive controls of data quality are carried out. The focus here is also on ensuring the protection of study participant data. At the RKI, a team from the internal quality assurance department 2 has been observing the study and quality assurance processes on a random basis. Among other measures, they examine the RKI study documents and conduct guideline-based interviews with senior RKI staff on relevant processes and corresponding quality assurance measures (e.g. in the RKI’s internal data management and laboratories). Internal reports are prepared and their results (i.e. observations and, if applicable, recommendations for action) are communicated to the study management at the RKI. These reports provide evidence of continued quality assurance.

### 2.3 Methods and survey contents

#### Laboratory analytics

In the examination part of the study, the collected dry blood specimens and mouth and nose swab samples are analysed in the laboratories at the RKI. The dry blood samples are punched out of the blood collection card and extracted before analysis. To determine the presence of IgG antibodies against the novel coronavirus, Euroimmun’s commercial laboratory test ‘Anti-SARS-CoV-2 ELISA (IgG)’ is used. With a sensitivity of 88.3% and a specificity of 99.4% (according to a validation study by the Paul Ehrlich Institute on reference sera, based on the analyses of 676 pre-pandemic samples and 222 convalescent COVID-19 patients, the vast majority (96%) of which were obtained at least 21 days after the onset of symptoms (personal communication from H. Scheiblauer, 25 September 2020)), the test is of high quality and has low cross-reactivity (Info box). The analyses are carried out in an automated process using Euroimmun’s high-throughput analyser ‘EUROLab Workstation ELISA’. According to the manufacturer, the dried blood samples are stable for 14 days at room temperature, and there is very high agreement between the dried blood and serum IgG measurement results (ratio: correlation coefficient 0.981; stability index 0.961. Positive versus non-positive result: sensitivity 100%; specificity 98.7% regarding serum results; n=215). A separate validation study on the agreement between serum and dry blood test results will be completed shortly.

Two different in-house PCR tests are performed in parallel on swab samples to detect the SARS-CoV-2 genome. Test 1 detects the E gene, adapted according to Corman et al. [18] and checks for possible errors in RNA extraction as well as possible PCR inhibition with a second PCR test conducted in parallel. Test 2 is specifically for SARS-CoV-2, targeting the ORF 1ab region of the viral genome, and can detect both the SARS-CoV-2 genome and cellular nucleic acids simultaneously, thus indicating successful sampling. If both individual tests are positive, the test as a whole is considered positive. In a methodology study, the two SARS-CoV-2 tests showed a very low detection limit; and, according to Corman et al [18], the specificity can be up to 100% at the laboratory analytical level. However, the relatively short time window in which viral material is detectable in the oral-nasal cavities of people infected with the virus, together with possible handling problems during sampling, mean that the actual sensitivity of the test is lower. A validation study with 103 self-swabs from adults and children showed a good level of acceptance and feasibility in regards to specimen collection. PCR test results for respiratory viruses compared well with the results from professionally collected swab specimens [19].

#### Survey data

The interview part of the RKI-SOEP study complements the laboratory analyses with a short interview of the study participants. A one-page paper questionnaire is filled out by each adult person in the household. The questionnaire asks questions about previous throat swab tests for SARS-CoV-2 and the results of these, the reasons for previous tests, the duration of any symptoms, and, if applicable, the course of a previous known SARS-CoV-2 infection and current symptoms.

The data obtained in the ‘CORONA-MONITORING bundesweit’ study (RKI-SOEP study) can also be linked to the data collected from participants in the regular survey waves of the SOEP, for example from the current year or previous years. In this way, demographic variables (e.g. age, household composition, region of residence), socioeconomic variables (e.g. education, occupation, income) as well as health-related variables (e.g. self-rated general health, chronic diseases, smoking, mental health) can be used in the RKI-SOEP study to analyse relevant risk and protective factors for a SARS-CoV-2 infection.

## 3. Expected results

### Statistical methods and estimation of sample size

On the basis of preliminary observations from group one in the gross sample, a 50% response rate is assumed for the entire sample by the end of the field period. This would correspond to a net sample size of about 15,837 adults. Assuming that 6% of biospecimens cannot be evaluated (an estimate based on empirical data from the first samples received), this gives a final net sample size of about 14,890 specimens for analysis.

The RKI-SOEP sample emerged from a complex study design. It is based (for the SOEP core samples) on households within multiple regional units spread across Germany and on the development of these households over the years. In addition, willingness to participate differs between the various population groups. For example, people with a migration background and those in full-time employment are less willing to participate than people who do not have a migration background or are not in full-time employment [20]. The study’s sampling error is therefore higher than if it had used a purely random sample, i.e. if individuals were drawn from a nationwide list and they all participated. To compensate for the resulting deviations from the total population, adjustment factors (sample weights) are calculated for the study and used in the analysis. The resulting increase in the sampling error can be roughly estimated using the so-called design effect [21]. The design effect for SOEP weighting factors is 2.8. This means that the expected sample size of about 15,840 persons corresponds to an effective sample size of about 5,320 persons, i.e. with regard to the sampling error it is comparable to a purely random sample of this size. Table 1 shows the expected case numbers and confidence intervals based on the effective sample size for different assumed seroprevalence levels in the population.

It is standard SOEP practice to use a variety of sociodemographic characteristics to derive the sample weights (see [13] for a detailed overview). In addition, the analyses of non-response in the present study consider the effect of the regional cumulative incidence of notified SARS-CoV-2 infections on people’s willingness to participate. Lastly, the study data are weighted by age, sex, as well as social and regional characteristics to match the German official population statistics (as of 31 December 2019) and the 2019 German Microcensus.

Weighted population estimates such as proportions of the population are used to estimate prevalence rates with regard to geographical area (federal state level, districts and urban districts). Survey procedures from established statistical programmes (SAS, Stata or R) are used, whereby the correlation within households as well as within municipalities is taken into account. Alternatively, confidence intervals are calculated using a clustered bootstrapping procedure at the 95% level.

Estimates are calculated for the following epidemiological indicators of infection, among others. The estimates are calculated for the total population and stratified by age group, sex, characteristics of socioeconomic status and other variables collected in the SOEP:

- Seroprevalence: the proportion of people who test positive for IgG antibodies (shown with and without adjustment for the sensitivity and specificity of the antibody test used)
- Degree of under-recording: the ratio of the frequency of SARS-CoV-2 infections estimated in the RKI-SOEP study to notified infections
- Extent of acute cases newly detected by the PCR test (oral-nasal swab).

Statistical associations with background factors (e.g. individual or regional characteristics) are assessed using weighted measures of association (such as odds ratios, average adjusted predictions or attributable risks from logistic regression models or Poisson models).

## 4. Discussion

The seroepidemiological ‘CORONA-MONITORING bundesweit’ study (RKI-SOEP study) combines the serological detection of SARS-CoV-2 antibodies in a nationwide sample of the adult population with PCR tests and interviews. This study design enables the identification of both past and current SARS-CoV-2 infections, including those that previously went undetected, which in turn helps to provide a more accurate estimation of the extent of the epidemic in Germany. The collected data on the number of people who have previously been infected also indicates the extent of under-recording of SARS-CoV-2 infections in the nationwide IfSG notification data.

A large number of antibody studies were initiated in Germany in 2020 [22, 23]. However, these studies have largely been conducted at a local or regional level, or are limited to specific settings such as companies, educational and healthcare institutions, or to population groups with above-average health. About one-third of these studies examine the spread of SARS-CoV-2 in the regional general population. Several studies have already reported results from municipalities that were particularly affected during the initial phase of the pandemic (see e.g. [24–27]). Initial results are also available from a Munich study as well as a study conducted in two urban areas in the city of Bonn [28, 29]. In addition, seroepidemiological studies are being conducted in the general population of selected regions [30] and in a nationwide cohort study of adults [31]. Numerous seroepidemiological studies have also been initiated internationally since March and increasingly since April 2020 [32–36]. Iceland [37], England [38], Brazil [39], Luxembourg [40], the Czech Republic [41] and Spain, among other countries, are conducting nationwide studies representative of their general populations [42].

The panel design of the RKI-SOEP study, i.e. that the same persons are surveyed every year, as well as the multidisciplinary nature of the SOEP survey programme are key to the study’s particular potential [12]. On the one hand, the panel design means that there is extensive information available on the participants from previous SOEP surveys, for example on their social situation and living conditions, which can be linked to RKI-SOEP study data. This gives the study the potential to be used more broadly, for example to analyse the social determinants of infection risks. Until now there have been hardly any data on the social determinants of infection risk, particularly at the level of individuals [43–45]. The RKI-SOEP study could therefore contribute to increasing knowledge in this area. On the other hand, the panel design also means that prospective data will be collected for years to come in the annual follow-ups of RKI-SOEP study participants within the regular and ongoing SOEP survey waves. The SOEP survey regularly includes topics such as health, illness, life satisfaction and wellbeing in addition to collecting data on people’s social situation, demographics and socioeconomic characteristics. The data could thus be used to compare social and health-related developments over the course of people’s lives depending on whether they were infected with SARS-CoV-2 during the pandemic or not. This in turn could provide insights into the long-term effects of a SARS-CoV-2 infection. One limitation of the study is the lack of clarity over how long IgG antibodies can be detected after infection, especially when the disease takes a mild course [46]. In addition, when drawing conclusions, the fact that antibody detection does not equate to immunity must be taken into account [47, 48].

In summary, the ‘CORONA-MONITORING bundesweit’ study (RKI-SOEP study) complements the antibody studies initiated previously, which so far have focused on specific population groups, with a nationwide study of the adult population. The study contributes toward obtaining a comprehensive picture of the spread of the virus in the population and in different population groups. It thereby also makes a contribution to an improved understanding of the SARS-CoV-2 epidemic in Germany, as well as to the identification of target groups for infection control measures.

## Key statements

- Seroepidemiological studies can contribute to an improved understanding of virus spread within the population.
- The RKI-SOEP study uses the infrastructure provided by the German Socio-Economic Panel (SOEP) to test people throughout Germany for past and present SARS-CoV-2 infections.
- Participants are asked to return a dry blood sample, an oral-nasal swab and a questionnaire.
- The samples are tested for SARS-CoV-2 IgG antibodies and SARS-CoV-2 RNA to identify past and present infections.
- The study can help provide a comprehensive picture of the nationwide spread of the virus as well as identify risk factors and protective factors for infection.

## Figures and Tables

**Table 1:** Expected precision for the seroprevalence estimates of infections with SARS-CoV-2 in the population of Germany aged 18 and older Source: Own table

Prevalence		Expected number of cases	Expected width of the 95% confidence interval
%			%
Total population	1.0	148	0.8–1.3
	2.0	297	1.7–2.4
	3.0	446	2.6–3.5
Population stratified into four equally sized age groups	1.0	37	0.6–1.7
	2.0	74	1.4–2.9
	3.0	111	2.2–4.1
